# Clinician-Patient Asynchronous Text Messaging Communication in Hospital-at-Home Care: Qualitative Study

**DOI:** 10.2196/83530

**Published:** 2026-06-08

**Authors:** Jeremy Soon Leong Seow, Stephanie Ko, Shi Yun Low, Shefaly Shorey

**Affiliations:** 1 Alice Lee Centre for Nursing Studies, Yong Loo Lin School of Medicine, National University of Singapore, Level 5, Centre for Translational Medicine, Block MD6, 14 Medical Drive, Singapore, 117599, Singapore, 65 66011294, 65 67767135; 2 Department of Medicine, Division of Advanced Internal Medicine, National University Hospital, Singapore, Singapore; 3 NUHS@Home, National University Health System, Singapore, Singapore

**Keywords:** hospital-at-home, digital communication, asynchronous communication, SMS text messaging, qualitative content analysis, patient-provider communication

## Abstract

**Background:**

Hospital-at-home (HaH) care models are increasingly being adopted as a strategy to treat older adults with acute care needs and reduce strain on health care systems. Technological innovations, particularly digital communication platforms, have become essential in enabling care delivery beyond traditional hospital settings. Among these, asynchronous messaging tools have the potential to facilitate safe, timely, and coordinated interactions between health care providers, patients, and caregivers. Despite growing interest, little is known about how the content and relational dynamics of such exchanges influence care experiences in real-world HaH contexts.

**Objective:**

This study aimed to examine the content of SMS text messaging exchanged between health care providers and patients or caregivers within a HaH program in Singapore during the COVID-19 pandemic.

**Methods:**

A descriptive qualitative design was used to analyze retrospective WhatsApp messages exchanged between health care providers and patients or caregivers from August 2022 to October 2022. An inductive qualitative content analysis approach was used to systematically identify and categorize emerging patterns in the data.

**Results:**

The analysis of 1218 WhatsApp messages from 354 HaH admissions identified three main categories: (1) clinical checks and advice; (2) administrative and transport arrangements; and (3) quality of interpersonal dynamics, supported by 13 subcategories, reflecting both task-oriented and relational dimensions of communication. The findings highlight how asynchronous messaging enables continuous care coordination while fostering trust and engagement between health care providers, patients, and caregivers.

**Conclusions:**

This study underscores the multifaceted role of digital communication in HaH care, demonstrating its influence beyond clinical coordination to operational efficiency and the quality of interpersonal relationships. The findings provide insights into how digitally mediated interactions supported care delivery and patient engagement within a Singapore HaH program during the COVID-19 pandemic. While situated within a pandemic-specific context, these findings offer transferable considerations for the design of communication strategies and digital tools to enhance the responsiveness, coordination, and patient-centeredness of HaH programs.

## Introduction

Globally, health care systems are facing increasing pressure due to constrained hospital bed capacity, rapidly aging populations, and a growing shortage of health care professionals [1]. These systemic challenges have driven interest in alternative care models that enhance capacity and improve care delivery beyond the conventional hospital environment, such as the hospital-at-home (HaH) care model, which delivers hospital-level care to patients within the comfort and familiarity of their home environment [2]. HaH programs are categorized into 2 main models: admission avoidance, which provides acute hospital-level care at home to prevent hospital admission, and early-supported discharge, which facilitates earlier discharge from the hospital with continued care delivered at home [3]. Systematic reviews have shown that HaH programs generally achieve clinical outcomes comparable to conventional inpatient care, while potentially reducing hospital length of stay and health care costs and improving patients’ and caregivers’ satisfaction [4,5].

During the COVID-19 pandemic, HaH programs were rapidly implemented in many health care systems to manage clinically stable patients in the community, while alleviating hospital bed pressures. Within HaH programs, technological advancements have further expanded the potential of these care models, with digital communication tools playing a pivotal role in enabling clinical care coordination and remote monitoring [6]. Health care services can be delivered more efficiently through eHealth modalities, such as mobile apps, digital communication platforms, and remote monitoring devices, further improving patient outcomes [7]. In HaH care models, digital communication platforms are commonly used to facilitate clinical communication, such as providing clinical updates and coordinating administrative concerns [8,9]. Effective communication among health care providers, patients, and caregivers is central to the success of HaH programs [10,11]. Unlike traditional inpatient care with routine face-to-face interactions, HaH relies on remote monitoring, virtual consultations, and periodic home visits to support timely information exchange [12]. These communication mechanisms are essential in maintaining patient safety, ensuring continuity of care, and facilitating real-time clinical decision-making in the home setting, effectively bridging hospital-level care within the home environment and strengthening the efficiency and viability of HaH programs [13,14].

While digital SMS text messaging has become a core component of communication in HaH programs, the actual content and dynamics of these interactions remain underexplored. Existing research has primarily focused on the operational benefits of digital tools, such as improving triage efficiency, facilitating clinical monitoring, and reducing hospital admissions, without fully examining how these platforms shape care relationships [15,16]. More recently, advances in artificial intelligence (AI)-driven conversational agents have further expanded the potential of digital health communication, with emerging studies examining the use of automated SMS text messaging systems and chatbots to support patient engagement and understand communication patterns in health care settings. However, comparatively little is known about the nature and dynamics of human-generated messaging interactions between health care providers and patients within HaH programs [17,18]. To address this gap, this study explored real-world SMS text messaging–based communication data generated during routine care between health care providers and patients or caregivers via WhatsApp, an asynchronous messaging platform that allows participants to send and receive messages without requiring real-time interaction to uncover patterns, themes, and tones. Beyond identifying informational content, it can also provide insights into how trust, empathy, and clinical authority are negotiated digitally, further illuminating insights into the relational labor embedded in HaH service delivery [19]. Therefore, understanding these digital dialogues is crucial for refining HaH models that are not only clinically effective but also relationally responsive and ethically grounded. As such, this study aimed to explore the content of SMS text messages exchanged between health care providers and patients or caregivers within the HaH program. Specifically, it addresses the research question: *What do the content and dynamics of digital text communication between health care providers, patients, and caregivers reveal about relational experiences in HaH programs?* The goal is for the findings of this study to inform the refinement of current and future HaH models, emphasizing the importance of digitally attuned communication practices and providing valuable insights to enhance health care provider training and strengthen patient engagement.

## Methods

### Study Design

A qualitative descriptive study design was used to explore the content of SMS text messages exchanged between health care providers and patients or caregivers within the HaH program. This design is appropriate as it contextually facilitates the grounded analysis of real-world SMS text message communications, offering insights into the content, tone, and relational dynamics that shape communication practices in real-world HaH settings [20]. The SRQR (Standards for Reporting Qualitative Research) checklist guided the reporting of this study [21] (Checklist 1).

### Sampling and Eligibility

A convenience sampling approach was adopted, drawing on all available deidentified SMS text message communications exchanged between health care providers, patients, and caregivers enrolled in the HaH program during the COVID-19 pandemic. Eligibility criteria included the following: (1) enrollment in the HaH program during the study period, (2) the availability of WhatsApp message records between the health care team and the patient or caregiver, and (3) SMS text messages written in English. All SMS text messages meeting these criteria and exchanged during the HaH admission period were included in the analysis. In routine care, WhatsApp communication occurred using the patient’s or caregiver’s own device.

### Setting

This study used retrospective data extracted between August and October 2022 from WhatsApp, a digital messaging platform used by health care providers and patients enrolled in the National University Health System (NUHS) program NUHS@Home. Established in 2020, NUHS@Home delivers hospital-level care at home for patients who would otherwise require inpatient hospitalization, primarily managing acute internal medicine conditions. During the study period, all patients included in this study were admitted as inpatient-level HaH cases and diagnosed with COVID-19, reflecting the patient population admitted at that time rather than a program-specific focus on COVID-19 care. In addition to SMS text messaging, patients received routine clinical monitoring through teleconsultations, remote monitoring, and home visits when clinically indicated. Patients who experienced clinical deterioration could be escalated for further evaluation or hospital admission according to established protocols. In this study, all included patients were managed entirely within the HaH setting and did not require escalation to inpatient hospitalization. As such, the dataset captures SMS text message communications during routine home-based care, and interactions related to acute deterioration may therefore be underrepresented.

WhatsApp was used by the clinical care team as part of routine clinical care, with patients agreeing to its use for service communication because of its high local adoption, support for group communication, and convenience. It was integrated into routine care as an asynchronous communication platform to facilitate both clinical and administrative communications between health care providers and patients or their caregivers. These uses included sharing clinical updates, monitoring symptoms, addressing patient queries, and coordinating logistical matters such as appointments and medication delivery.

The study team comprised an internal medicine clinician (SK), a registered nurse (JSLS), a doctoral-trained researcher (SS), and a public health researcher (SYL) with formal training and experience in qualitative methodology. The diversity of professional backgrounds offers a multidisciplinary lens, further enriching the interpretation and contextualization of communication dynamics within the HaH setting.

### Ethical Considerations

The study received ethical approval from the National Health Group Domain-Specific Review Board (reference 2021/00896), which serves as the ethics board of the participating institution. Specific research consent was waived by the ethics board because this study involved a retrospective analysis of deidentified routine care data. To ensure anonymity and confidentiality, data from the participants were deidentified and assigned coded pseudonyms.

### Data Collection

Qualitative data were retrospectively extracted from WhatsApp comprising all SMS text messages exchanged between the NUHS@Home team and patients or caregivers between August 2022 and October 2022. Demographic characteristics of patients (eg, age, sex, and ethnicity) were obtained from the electronic medical records to describe the study population. Sample messages included conversations related to episodes of care or administrative coordination. As this was an exploratory retrospective qualitative study, no a priori sample size was specified. All eligible messages exchanged during the study period were included in the analysis. During iterative coding, category development was considered sufficiently comprehensive when no substantially new patterns or insights emerged from further review of the dataset [22].

### Rigor

Rigor was established using the guidelines of credibility, transferability, dependability, and confirmability developed by Lincoln and Guba [23]. Credibility was enhanced through investigator triangulation during data analysis, which involved interdisciplinary team members interpreting the data. This ensured that multiple perspectives were incorporated and findings were grounded in the evidence. Transferability was maintained by providing detailed contextual descriptions of the setting and participant characteristics, allowing readers to assess relevance to other care environments. Dependability was ensured through the comprehensive documentation of an audit trail, comprising raw SMS text message data, coding, and analytic processes, to provide transparency and consistency. Confirmability was enhanced through reflexive journaling and independent coding by 2 researchers, followed by discussion and consensus-building on coding differences. As emphasized by Teh and Lek [24], reflexivity is a vital component of qualitative research that helps to mitigate unconscious researcher bias. The reflexive journal enabled the research team to critically examine their assumptions and decision-making, thereby enhancing the overall trustworthiness of the study [25].

### Data Analysis

Content analysis was used to explore the SMS text messages exchanged between health care providers and patients or caregivers within the HaH program. This method was selected for its established applicability in analyzing communication and media-based data, making it well-suited to the study’s focus on clinical SMS text messaging [26]. Content analysis allows researchers to interpret contextual meaning through a systematic coding process, thereby uncovering patterns and themes within communication [27]. Two members of the research team (SS and JSLS), both trained in qualitative research methods, independently reviewed and coded the data. Discrepancies were addressed through collaborative discussions among the research team, ensuring that the findings were accurately represented. Microsoft Word was used to organize and manage the dataset during the coding process. The 3-step inductive approach proposed by Elo and Kyngäs [28] guided the analytical process. In the preparation phase, researchers immersed themselves in the dataset by reading the messages several times to gain familiarity and determine appropriate units of analysis. Message timestamps were used at this stage to establish the chronological sequence of exchanges and to reconstruct conversational threads between health care providers and patients or caregivers. This enabled the identification of message initiation and response patterns within each interaction. In the organizing phase, the data were segmented into meaning units and coded to capture key content. These codes were then grouped into categories and subcategories through a process of abstraction. Finally, in the reporting phase, these categories were refined to reflect the recurring patterns present in the data.

## Results

### Overview

From August 2022 to October 2022, a total of 354 HaH admissions met the study inclusion criteria and were included in the analysis. All included patients were managed within the HaH settings without requiring inpatient hospitalization, and all patients used asynchronous communication via WhatsApp. The mean age of the participants was 57 (SD 17.8) years. Most study participants were female (199/354, 56.2%), and the group was composed predominantly of individuals with Chinese ethnic backgrounds (246/354, 69.5%; Table 1). The primary diagnosis was COVID-19 (N=354), and the mean length of stay in HaH was 8 (SD 4) days. A total of 1218 SMS text messages were examined and coded from the conversations between health care providers and patients or caregivers. The median number of WhatsApp messages exchanged between the care team and patients or caregivers per patient interaction was 8 (IQR 13.5). Among the coded messages, 61.1% (364/596) were provider-initiated and 38.9% (232/596) were patient- or caregiver-initiated, based on the initiation of conversational threads. The median response time for health care providers was 11 (IQR 21.5) minutes during office hours (8 AM-6 PM) and 3 (IQR 6.25) minutes after office hours (6 PM).

**Table 1.** Sociodemographic, clinical, and SMS text messaging^a^ characteristics of patients (N=354).

Collected data	Values
Age (y), mean (SD)	57 (17.8)
Male, n (%)	155 (43.8)
Ethnicity, n (%)	
Chinese	246 (69.5)
Indian	29 (8.2)
Malay	42 (11.9)
Others	37 (10.5)
Primary diagnosis, n (%)	
COVID-19	354 (100)
Length of stay in hospital-at-home (d), mean (SD)	8 (4)
Message thread per patient interaction, median (IQR)	8 (13.5)
Message directionality (n=596), n (%)	
Provider-initiated	364 (61.1)
Patient or caregiver-initiated	232 (38.9)
Response time for health care providers during office hours (8 AM-6 PM; min), median (IQR)	11 (21.5)
Response time for health care providers after office hours (after 6 PM; min), median (IQR)	3 (6.25)

a Total number of messages=1218.

Three categories were identified from the analysis of the SMS text messages, reflecting the nature of interactions between the health care providers and patients or caregivers within the HaH program: (1) clinical checks and advice, (2) administrative and transport arrangements, and (3) quality of interpersonal dynamics. These categories were further supported by 13 subcategories (Textbox 1).

Textbox 1. **Categories and subcategories identified from the content analysis.**

**Category 1: clinical checks and advice**

- Clinical consultation
- Vitals monitoring by health care providers
- Vitals monitoring by patients and caregivers
- Medication-related inquiries

**Category 2: administrative and transport arrangements**

- Logistical support and transport coordination
- Administrative transition
- Establishing lines of communication

**Category 3: quality of interpersonal dynamics**

- Emotional support
- Appreciation
- Trust and feedback
- Courteous communication
- Communication timeliness
- Tone and professional etiquette

### Category 1: Clinical Checks and Advice

#### Overview

A total of 339 coded SMS text messages were categorized under clinical checks and advice, which reflected a range of provider-patient communications focused on monitoring and maintaining the clinical stability of patients in HaH. These were further supported by 4 subcategories: clinical consultation (91 SMS text messages), vitals monitoring by health care providers (157 SMS text messages), vitals monitoring by patients and caregivers (33 SMS text messages), and medication-related inquiries (58 SMS text messages). These subcategories were further elaborated through detailed codes as outlined in the coding framework in Table 2 (see Multimedia Appendix 1 for coding scheme).

**Table 2.** Coding framework for clinical checks and advice category (SMS text messages for coding: n=339).

Subcategories and code	SMS text messages for subcategory coding, n (%)	SMS text messages for code coding, n
Clinical consultation	91 (26.8)	
Family member seeks clinical advice from health care provider		55
A health care provider gives clinical advice		8
Updates on tests		28
Vitals monitoring by health care providers	157 (46.3)	
Health care provider following up on patient’s abnormal vitals		54
Health care provider instructs on vital monitoring		103
Vitals monitoring by patients and caregivers	33 (9.7)	
Patient or caregiver informs of patient’s vitals		33
Medication-related inquiries	58 (17.1)	
Patient or caregiver clarifies about medication		19
Health care provider instructs on administering medication		31
Health care provider clarifies the patient’s or caregiver’s medication request		8

#### Clinical Consultation

Three codes were used to categorize 91 SMS text messages related to clinical consultations, which reflected patients or caregivers seeking medical input, health care providers offering clinical advice, and both parties exchanging updates on tests. These SMS text messages often reflected patients or caregivers who sought clarification from health care providers regarding their symptoms and ongoing health concerns, particularly in situations where they were uncertain about the appropriate course of action:

*Hi my mum is complaining about severe pain at her neck. Do you advise us to [go to] an [accident and emergency department] A&E or [is there] any doctor to review her?*

[Caregiver A]

*This [sharing a photo] is the diaper with blood stains, seems to be from the penile area [and the] catheter also shows some blood clots, should we be concerned?*

[Caregiver B]

Furthermore, health care providers offered clinical guidance on the symptoms and overall well-being of patients and also interpreted and communicated diagnostic test results to patients and their caregivers:

*[Team] wanted to let you know that the blood results were good [and] we are planning to drop by tomorrow to remove the PICC [Peripherally-Inserted Central Catheter]*

[Doctor A]

*Hi Madam [patient], the results from today’s labs show [that] your magnesium is a little low today, so tomorrow morning we will top up with some IV [Intravenous] magnesium*.

[Doctor A]

#### Vitals Monitoring by Health Care Providers

Two codes were used to categorize 157 SMS text messages related to vital signs monitoring by health care providers, which included active tracking and review of patients’ parameters such as blood pressure, temperature, oxygen saturation, and glucose levels. These SMS text messages commonly included reminders to submit readings, follow-up assessments based on the submitted data, and clinical interpretations shared with patients:

*Can you help to recheck the blood pressure in 15 mins again? It is very high now.*

[Doctor A]

*Mr [patient], I would like to update you that from tomorrow onwards, you only need to submit your vital signs once in the morning will do*.

[Doctor C]

#### Vitals Monitoring by Patients and Caregivers

Thirty-three SMS text messages were categorized into one code related to vital signs monitoring conducted by patients and caregivers. These messages captured instances where patients or their caregivers independently initiated the reporting of vital parameters, such as blood pressure, temperature, and oxygen saturation, via SMS text messages:

*Glucose test after breakfast: 7.3 mmol/l.*

[Patient B]

#### Medication-Related Inquiries

Three codes were used to categorize 58 SMS text messages related to medication-related inquiries. These messages reflected questions from patients or caregivers regarding various aspects of medication management, such as prescribed regimens, timing of administration, and potential side effects:

*May I know can I continue my Lenalidomide this evening?*

[Patient C]

*Can [patient] take paracetamol for fever/headaches or not advisable due to chemo.*

[Caregiver C]

Similarly, health care providers also provided medication guidance, such as clarification of prescribed drugs and adjustments to dosages based on symptoms reported by patients:

*Good morning Ms [patient]. I am the nurse whom spoke to [you] earlier [and] regarding the flumucil, there will not be an issue for you to take.*

[Nurse A]

[You] *can put ketoprofen plaster, [it] needs to be changed every 12 hourly…*

*[Doctor A*]

### Category 2: Administrative and Transport Arrangements

#### Overview

A total of 213 coded SMS text messages were categorized under administrative and transport arrangements, primarily reflecting logistical coordination, administrative processes, and efforts to establish effective communication between the health care team, patients, and caregivers. These were further supported by 3 subcategories: logistical support and transport coordination (42 SMS text messages), administrative transition (95 SMS text messages), and establishing lines of communication (76 SMS text messages). These subcategories were further elaborated through detailed codes as presented in the coding framework in Table 3.

**Table 3.** Coding framework for administrative and transport arrangements category (SMS text messages for coding: n=213).

Subcategories and code	SMS text messages for subcategory coding, n (%)	SMS text messages for code coding, n
Logistical support and transport coordination	42 (19.7)	
Arrangement for delivery/pick-up of logistics		36
Transport arrangement for patients		6
Administrative transition	95 (44.6)	
Discharge arrangements		35
Arrangements made for appointments and follow-up		42
Request for documentation		18
Establishing lines of communication	76 (35.7)	
Health care provider checks the contact availability of the patient or caregiver		19
Establishing a point of contact for the patient		26
Scheduling for a home visit		31

#### Logistical Support and Transport Coordination

Two codes were used to categorize 42 SMS text messages related to logistical support and transport coordination within the HaH model. The messages captured included arrangements for the delivery of medical devices and the replenishment of consumables, which were crucial to ensuring efficient care delivery:

*Mdm [patient], what time can we send over the nurse box to your house [as] we need someone to be home to receive it?*

[Doctor A]

Additionally, transport coordination emerged as a vital component, with messages detailing the planning and organization of ambulance services for diagnostic procedures or transitions between care settings:

*Hi, the team is planning for Mdm [patient] to return to NUH for dressing change for the tunnelled catheter. We will be booking [the] transport, kindly standby for the timing*.

[Doctor A]

#### Administrative Transition

Ninety-five SMS text messages were categorized into 3 codes related to administrative processes within the HaH model. These SMS text messages often involved a range of operational and clerical activities, such as the coordination of patient admission and discharge procedures, the completion of documentation requirements, and financial matters:

*Could you provide me your email so we can send you the discharge documents?*

[Doctor A]

*Can we have [a] document [to] show [that] my father was warded at home for this period?*

[Caregiver D]

#### Establishing Lines of Communication

Three codes were used to categorize 76 SMS text messages capturing efforts to initiate or clarify communication among patients, caregivers, and health care providers. These SMS text messages often included providing contact details, verifying preferred communication channels (eg, text and call), and scheduling home visits:

*Hi Mdm [patient], let me know when you [are] ready [for us to call]*.

[Doctor A]

*I am the granddaughter of Mr [patient]. You may reach out if you need anything*.

[Caregiver E]

*Team, just to check will the nurse come today?*

[Patient D]

### Category 3: Quality of Interpersonal Dynamics

#### Overview

A total of 666 SMS text messages were categorized under interpersonal communication quality, emphasizing the central role of relational dynamics and the multifaceted nature of interpersonal interactions within the HaH care model. These were further supported by 6 subcategories: emotional support (14 SMS text messages), appreciation (11 SMS text messages), trust and feedback (10 SMS text messages), courteous communication (48 SMS text messages), communication timeliness (10 SMS text messages), and tone and professional etiquette (573 SMS text messages). These subcategories were further elaborated through codes as presented in the coding framework in Table 4.

**Table 4.** Coding framework for the quality of interpersonal dynamics category (SMS text messages for coding: n=666).

Subcategories and code	SMS text messages for subcategory coding, n (%)	SMS text messages for code coding, n
Emotional support	14 (2.1)	
The health care provider provides emotional support to patients or caregivers		14
Appreciation	11 (1.7)	
Patients or caregivers showing appreciation		11
Trust and feedback	10 (1.5)	
Patient feels comfortable sharing concerns with the health care provider		6
Negative feedback		4
Courteous communication	48 (7.2)	
Patient or caregiver positively acknowledges instructions		48
Communication timeliness	10 (1.5)	
Delayed reply from patients or caregivers		2
Delayed reply from health care providers		4
Health care provider responds to patient’s missed call		4
Tone and professional etiquette	573 (86)	
Informal tone by patient or caregiver		163
Formal tone by patient or caregiver		94
Informal tone by health care provider		189
Formal tone by health care provider		127

#### Emotional Support

Fourteen SMS text messages were categorized into 1 code, reflecting expressions of empathy, reassurance, and encouragement conveyed by health care providers through SMS text messages:

*Do not worry about the NUCOT [National University Centre for Organ Transplantation] blood test, we will do the necessary labs at home during this admission*.

[Doctor A]

#### Appreciation

Eleven SMS text messages were categorized into 1 code, reflecting gratitude from patients or caregivers for acknowledging the health care team’s dedication, responsiveness, and compassion:

*Hi doctor and medical team, thank you for very much for taking care of me.*

[Patient E]

#### Trust and Feedback

Two codes categorized 10 SMS text messages related to expressions of trust and the exchange of feedback between patients, caregivers, and health care providers. These text messages reflected the growing confidence of patients in the clinical decisions made on their behalf and their willingness to voice concerns, ask questions, and engage in open dialogue:

*My blood pressure [is] very high, 179/113 [mmHg]. I am worried*.

[Patient F]

In addition to positive affirmations, a smaller number of messages conveyed dissatisfaction with aspects of care delivery. These instances of negative feedback highlighted areas where expectations were unmet and served as critical input for service improvement and patient-centered responsiveness in home-based care:

*It is not [a] problem for me to continue [to] submit the vitals. However, I need a clear direction from your side. The direction does not [seem to be] clear [and] now it’s just submission of vital, if [give] wrong medication, how?*

[Patient G]

#### Courteous Communication

Forty-eight SMS text messages were categorized into 1 code reflecting the consistent use of courteous language, such as expressions of gratitude and polite message closures exchanged among patients, caregivers, and health care providers. Patients and caregivers frequently acknowledged and affirmed the guidance provided by health care professionals, demonstrating attentiveness and appreciation:

*Thanks a lot and noted on this*.

[Patient H]

*I am thankful to have the [healthcare] team [and] to benefit from the hospital-at-home programme, [it was] very helpful to me and my family.*

[Patient I]

#### Communication Timeliness

Ten SMS text messages were categorized into 3 codes that addressed the timeliness of responses exchanged between patients, caregivers, and health care providers within digital communication. These codes captured a subset of messages highlighting delayed responses related to administrative matters after standard office hours (6 PM), involving both patients and health care providers:

*Hi Mr [patient], awaiting your email address*.

[Doctor A]

*Sorry to revert back so late, we will discharge you tomorrow as planned.*

[Doctor A]

In some instances, calls initiated by patients were not immediately answered by health care providers. Nevertheless, health care providers consistently made efforts to follow up and respond as soon as possible:

*Hi Mr [patient], we got a missed call from you. Is there anything we can help you?*

[Doctor A]

#### Tone and Professional Etiquette

A total of 573 SMS text messages were categorized into 4 codes related to the tone of communication exchanged between patients, caregivers, and health care providers in digital messaging. The tone varied according to context, with both formal and informal language observed across interactions. A formal tone was predominantly used in messages concerning clinical guidance, updates, or health-related discussions, demonstrating professionalism and clarity in clinical communication. In contrast, an informal tone was more common in exchanges regarding administrative matters such as scheduling or logistics, suggesting a more conversational and approachable style suited to routine coordination.

*Good morning, would like to remind [you] to submit today’s morning vitals please. Thank you*.

[Doctor A]

*Hi [doctor], my dad agrees to the infusion. Please let me know the arrangements once you have the details, many thanks*.

[Caregiver F]

*Hi! Btw can you please help submit the consent form again but this time in your dad’s name? haha so sorry thank you!*

[Doctor C]

## Discussion

### Principal Findings

This study examined the content of SMS text messages exchanged between health care providers and patients or caregivers within a HaH program in Singapore, offering insights into clinical communication and the relational dynamics enabling remote care delivery. Although the findings were derived from a cohort composed entirely of patients with COVID-19 during August 2022 to October 2022 and comprised low-acuity cases with no escalation to inpatient care, they offer potentially transferable insights into asynchronous communication processes within HaH settings. However, their applicability to other diagnoses should be interpreted cautiously. To our knowledge, this is the first content analysis of asynchronous communications within a HaH service in Singapore.

Communication related to the clinical aspects of care emerged as a key category in text messages exchanged between patients, caregivers, and health care providers within the HaH program. These interactions were centered on clinical consultations, where patients and caregivers sought medical advice related to symptom management, medication clarification, and general health concerns. In parallel, health care providers used these messages to deliver clinical updates, offer guidance, and adjust care plans based on patient conditions. This closely aligns with studies conducted in the United States and China, suggesting that SMS text messaging–based communication between clinicians and patients frequently involved symptom discussions, disease management support, and medication concerns [29-31]. Additionally, frequent SMS text message exchanges related to the monitoring of clinical parameters, such as reminders to report vital sign readings initiated by both health care providers and patients or caregivers, were observed. These were often followed by health care providers’ interpretations of the submitted data, which subsequently guided further clinical decision-making. This communication pattern aligns with findings from a systematic review, which emphasized that remote vital signs monitoring is a foundational element of HaH programs, facilitating the early detection of clinical deterioration and timely intervention when necessary [32]. Collectively, these observations underscore the value of asynchronous communication as a critical enabler of clinical continuity in home-based care. Through timely and responsive digital communications, it bridges the gap between patients and health care providers, allowing for ongoing monitoring, real-time decision support, and patient engagement that aligns with the core principles of the HaH model. As such, future research may benefit from examining how the frequency and quality of digital clinical communication can influence patient outcomes, such as symptom resolution and overall satisfaction with care.

Administrative and transport arrangements were identified as another key category in this study, highlighting the importance of nonclinical operational components in enabling effective care delivery within the HaH model. Numerous SMS text messages reflected the coordination of logistical tasks and administrative and transport-related processes. These interactions included scheduling home visits, organizing the delivery of medications and medical consumables, and facilitating transitions between care settings, such as hospital discharge to home or readmission when clinically indicated. Moreover, administrative communications reflected efforts to clarify documentation requirements, streamline admission and discharge workflows, and address patients’ or caregivers’ queries related to billing. These findings are consistent with studies conducted in the United States and the Netherlands, suggesting that digital messaging platforms are frequently used to manage a wide range of clerical and logistical tasks, such as administrative coordination, logistical planning, and transport arrangements [33,34]. When effectively streamlined, these processes can contribute to a reduction in delays in care delivery and improved patient experiences within remote care settings [35]. Interestingly, qualitative studies conducted in Singapore have highlighted that logistical and operational hurdles are inevitable in the implementation of HaH programs, potentially disrupting care continuity and resulting in delays, increased health care provider workload, and reduced patient satisfaction [36,37]. Therefore, addressing these issues is essential to optimize the effectiveness and scalability of HaH services in Singapore. Furthermore, the establishment of clear and reliable communication pathways between health care teams and patients or caregivers was observed. These interactions included identifying preferred modes of communication, whether via SMS text messaging or telephone calls, and designating a consistent point of contact for the care team. Similarly, this is consistent with a study conducted in the United States, highlighting the importance of aligning communication channels with patient preferences to enhance engagement and satisfaction [38]. Thus, clarifying communication preferences and responsibilities not only fosters continuity of care but also supports timely responsiveness and reduces fragmentation in care delivery. All in all, these findings underscore the integral role of digital communication in supporting the administrative and logistical infrastructure of HaH models. Therefore, stakeholders may consider engaging in co-design processes with end users of HaH programs to develop a tailored digital application aimed at streamlining operational workflows, further alleviating the administrative burden on health care teams while enhancing the overall patient experience and satisfaction.

Another key category identified in this study was the quality of interpersonal dynamics reflected in the digital text message exchanges between the health care providers, patients, and caregivers. These interactions consisted of expressions of empathy and emotional reassurance from health care providers and words of appreciation and gratitude from patients and caregivers in response to the care they received. Notably, although these interactions reflected elements of emotional support, explicit discussions of mental health conditions were not observed. This may reflect the clinically oriented nature of routine HaH communication, the likelihood that sensitive psychosocial concerns were addressed through alternative channels (eg, through synchronous audio or video calls), or a reluctance to disclose mental health issues within an SMS text messaging–based clinical exchange [39,40]. Furthermore, respectful and courteous language was observed across both parties, reinforcing the role of digital communication in fostering trust and mutual respect. These findings are consistent with multisite studies conducted in Australia, Ireland, the United Kingdom, Spain, and Germany, which similarly observed empathetic communication, emotional support, and courteous exchanges within digital platforms [40,41]. Moreover, the presence of digital empathy within asynchronous SMS text messaging–based interactions has been shown to foster rapport, encourage participant engagement, and contribute to positive patient experiences in HaH models [42]. Such relational interactions can potentially help to humanize remote care delivery and are instrumental in cultivating therapeutic alliances, which may positively influence patient engagement, satisfaction, and perceived quality of care. Furthermore, with the contemporary workforce gravitating toward asynchronous communication, professional environments have widely integrated digital tools to facilitate collaboration and optimize team productivity [43]. Future research could explore how these relational dynamics shape the experience of patients, caregivers, and health care providers, which could inform the development of culturally sensitive communication frameworks and targeted training programs for health care providers that will support high-quality relational care in digitally mediated HaH settings.

Timeliness of communication and professional tone were identified as another key category in the SMS text message exchanges between health care providers, patients, and caregivers in this study. Several interactions revealed delayed responses to nonclinical inquiries, particularly those sent after standard office hours. This finding aligns with studies conducted in China and the United States, which also reported frequent response delays after office hours [29,44]. Notably, timely communication has been shown to significantly influence patient satisfaction, likely due to its role in reinforcing feelings of safety, emotional reassurance, and trust, which are essential qualities valued in HaH models of care [44,45]. In addition to timeliness, the tone of communication provided further insights into the relational dynamics between health care providers, patients, and caregivers. Our findings highlighted that a formal tone was often used in messages on clinical care or medical guidance, reflecting a focus on clarity and professionalism. In contrast, informal tones were more frequently observed in administrative or logistical messages, indicating a conversational style. These patterns are congruent with studies conducted in the United States, which highlighted that clinically significant messages were communicated in a more formal tone, while operational matters were addressed in a neutral or informal tone [46,47]. Together, these findings suggest that tone modulation according to message purpose may play an important role in balancing clinical professionalism with relational rapport in HaH care. Future research could explore how tone preferences vary across cultural and demographic groups and examine how tone influences perceived empathy, patient engagement, and satisfaction within digital health care communication.

### Implications of Future Practice and Research

Our findings suggest several recommendations to strengthen digital communication practices within HaH care. First, 2-way asynchronous communication among patients, caregivers, and providers appears to be an important enabler of HaH care, helping patients and caregivers feel connected and supported, while allowing the care team to convey information efficiently. Second, the use of an existing platform that patients and caregivers are highly familiar with is critical to optimize their responsiveness and experience. Third, guidelines for asynchronous communications for HaH teams will be helpful to optimize clinical effectiveness and patient engagement. This may include types of content suitable for asynchronous communication (eg, instructive or informative rather than discussive), language and tone (eg, professional yet friendly), target response times, and after-hour response expectations. Dedicated training may be necessary for health care providers on the effective use of asynchronous communication tools as their use expands. Understanding the nature of these communications also has implications on the development of future AI-assisted communication tools that can be trained to answer such queries.

Future research directions can explore differences in communication among older adults and how SMS text messaging–based relational elements, such as empathy and mutual respect, shape the experiences of patients and caregivers. Intervention-focused studies could build on these findings using theory-informed approaches. Further work could also examine how tone preferences vary across cultural or demographic groups. Our findings also suggest a need for digital platforms that are better tailored to the operational realities of HaH care to streamline administrative tasks and reduce the burden on health care teams. Future research could also explore how generative AI technologies, such as large language models and conversational agents, could augment SMS text messaging–based communication within HaH programs by supporting routine queries and summarizing message threads. Nevertheless, given the central role of empathy and relational dynamics identified in this study, careful consideration will be critical to ensure that such innovations support the human dimensions of care. These offer valuable opportunities to inform more patient-centered, culturally attuned, and operationally effective communication strategies that support high-quality HaH care.

### Limitations

This study has several limitations. First, the dataset primarily comprised English-language communications, which may not fully capture the experiences of non-English-speaking participants. This limits the inclusivity and cultural representativeness of the findings, particularly in a multilingual context like Singapore. Additionally, the sample did not adequately reflect the broader demographic trends of an aging population, who are key users of the HaH services. Hence, future studies may benefit from purposive sampling strategies that include a more diverse linguistic and age profile, such as older adults, to provide a more comprehensive understanding of digital communication in HaH care. Second, the retrospective nature of the SMS text message data limits insights into the broader context of communication exchanges, as asynchronous messages may not have fully captured the full scope of interactions, particularly those that occurred following or preceding a verbal or face-to-face encounter. As such, future studies may consider triangulating message data with complementary methods, such as semistructured interviews or ethnographic observations, to better contextualize communication dynamics. Third, due to the time period of sampling, all patients were admitted for COVID-19. However, although remote monitoring, home visits, and virtual consultations are also used in other HaH diagnostic groups, communication patterns may differ by condition severity, symptom profile, and care trajectory. Therefore, transferability to a broader HaH population should be interpreted with caution. Finally, although qualitative content analysis was used to derive insights, the potential for researcher bias remains inherent due to the interpretative process. To mitigate this, systematic coding procedures and regular team discussions were used within the research team.

### Conclusion

This study offered valuable insights into the nuances of digital communication between health care providers, patients, and caregivers within a HaH program in Singapore, in the context of the COVID-19 pandemic. The findings underscore the critical interplay between operational efficiency, the quality of interpersonal relationships, and clinical effectiveness in shaping the overall success of digitally mediated care within HaH settings. These interconnected elements not only facilitate seamless care coordination but also foster patient trust, engagement, and overall satisfaction. These insights underscore the need for communication frameworks and training programs that are clinically robust, operationally efficient, and attuned to the relational dynamics essential for high-quality home-based care. As HaH models continue to expand, future research should integrate these communication principles into health care provider training, digital platform development, and implementation strategies to promote more person-centered, culturally responsive, and sustainable care delivery.

## Supplementary material

10.2196/83530 Multimedia Appendix 1 Coding scheme of text messages

10.2196/83530 Checklist 1 SRQR checklist.

## Abbreviations

AI: artificial intelligence
HaH: hospital-at-home
NUHS: National University Health System
SRQR: Standards for Reporting Qualitative Research
