# Supplementary material for: Clinician-Patient Asynchronous Text Messaging Communication in Hospital-at-Home Care: Qualitative Study
Source: J Med Internet Res. 2026 Jun 8;28:e83530. doi: 10.2196/83530 (PMC13245894; doi:10.2196/83530)
Supplement: Multimedia Appendix 1 [file jmir-v28-e83530-s001.docx]

**Appendix: Coding scheme of text messages**

| Code | Code definition | Example |
| --- | --- | --- |
| Clinical checks and advices | | |
| Family member seeks clinical advice from healthcare provider | Family member seeks guidance from a nurse/doctor regarding patient’s symptoms when unsure of the appropriate course of action | ‘Hi my mum is complaining about severe pain at her neck. Do you advise us to [go to] an [accident and emergency department] A&E or [is there] any doctor to review her?’ [Caregiver A]  ‘This is the diaper with blood stains, seems to be from the penile area [and the] catheter also shows some blood clots, should we be concerned?’ [Caregiver B] |
| Healthcare provider gives clinical advice | Healthcare provider responds to concerns raised by the caregiver/patient regarding the patient’s symptoms and well-being | ‘Hi [patient], I have passed your previous gel dressing to the speedoc nurse who is on the way to your house now and asked him to check on the [peripherally inserted central catheter] PICC site as well.’ [Doctor A]  ‘[Team] wanted to let you know that the blood results were good [and] we are planning to drop by tomorrow to remove the PICC’ [Doctor A] |
| Updates on tests | Healthcare provider updates the patient/caregiver about blood test or urine culture results (or vice-versa) | ‘Hi Madam [patient], the results from today’s labs show [that] your magnesium is a little low today, so tomorrow morning we will top up with some IV magnesium.’ [Doctor A] |
| Healthcare provider following up on patient’s abnormal vitals | Healthcare provider follows up when patient’s submitted vitals indicate a concern | ‘Can you help to recheck the blood pressure in 15 mins again? It is very high now.’ [Doctor B] |
| Healthcare provider instructs on vitals monitoring | Healthcare provider informs patient/caregiver about the updated frequency of vital signs monitoring | ‘Mr [patient], I would like to update you that from tomorrow onwards, you only need to submit your vital signs once in the morning will do.’ [Doctor C] |
| Patient/caregiver informs of patient’s vitals | Patient/caregiver submits patient’s vital signs (temperature, oxygen saturation, blood pressure or blood sugar levels) | ‘Glucose test after breakfast: 7.3.’ [Patient B] |
| Patient/caregiver clarifies about medication | Patient/caregiver seeks advice about medications | ‘May I know can I continue my Lenalidomide this evening?’[Patient C]  ‘Can [patient] take paracetamol for fever/headaches or not advisable due to chemo’ [Caregiver C] |
| Healthcare provider instructs on administering medication | Healthcare provider provides the patient/caregiver with instructions on medications usage | ‘Good morning Ms [patient]. I am the nurse whom spoke to [you] earlier [and] regarding the flumucil, there will not be an issue for you to take.’ [Nurse A]  ‘[You] can put ketoprofen plaster, [it] needs to be changed every 12 hourly...’ [Doctor A] |
| Healthcare provider clarifies patient’s/caregiver’s medication request | Patient requests for medication and healthcare provider clarifies type/name of medication needed | ‘Good morning Mr [patient], I am [the nurse whom] spoke to you earlier. Sorry, could you let me know the name of the nose spray again so I can tell the pharmacist.’ [Nurse B] |
| Administrative and transport arrangements | | |
| Arrangement for delivery/pick-up of logistics | Delivery of telemonitoring equipment and medications | ‘Mdm [patient], what time can we send over the nurse box to your house [as] we need someone to be home to receive it?’ [Doctor A] |
| Transport arrangement for patients | NUHS@Home arranges transportation for the patient to and from the hospital for appointments | ‘Hi, the team is planning for Mdm [patient] to return to NUH for dressing change for the tunnelled catheter. We will be booking [the] transport, kindly standby for the timing.’ [Doctor A] |
| Discharge arrangements | Administrative matters prior to discharge | ‘Could you provide me your email so we can send you the discharge documents?’ [Doctor A] |
| Arrangements made for appointments and follow-up | Scheduling of conflicting outpatient appointments | ‘Hi, please be informed that my mum has a medical appointment at Novena medical at 2pm. Please arrange for IV slot earlier, thanks.' |
| Request for documentation | Patient/caregiver requesting for hospital leave or medical certificate | ‘Can we have [a] document [to] show [that] my father was warded at home for this period?’ [Caregiver D] |
| Healthcare provider checks contact availability of patient/caregiver | Healthcare provider confirms the availability of the patient/caregiver before making the phone call | ‘Hi Mdm [patient], let me know when you [are] ready.’ |
| Establishing point of contact for patient | Patient/caregiver discusses with the healthcare provider the contact details of the person to be reached during admission | ‘I am the granddaughter of Mr [patient]. You may reach out if you need anything.’ [Caregiver E] |
| Scheduling for home visit | Healthcare provider liaising with patient (and vice versa) on upcoming face-to-face home visit | ‘Team, just to check will the nurse come today?’ [Patient D] |
| Quality of interpersonal dynamics | | |
| Healthcare provider provides emotional support to patients/caregivers | Healthcare providers offers reassurance to patients/caregivers regarding the current situation causing concern | ‘Do not worry about the NUCOT blood test, we will do the necessary labs at home during this admission.’ [Doctor A] |
| Patients/caregivers showing appreciation | Patient/caregiver showing appreciation for NUHS@Home team | ‘Hi doctor and medical team, thank you for very much for taking care of me.’ [Patient E] |
| Patient feels comfortable sharing concern with healthcare provider | Patients feel comfortable with healthcare provider and express their concerns | ‘My blood pressure [is] very high, 179/113. I am worried.’ [Patient F] |
| Negative feedback | Patient/caregiver expresses dissatisfaction with service provided | ‘By the way, It is considered impolite to spell surname in small cap.’  It is not [a] problem for me to continue [to] submit the vitals. However, I need a clear direction from your side. The direction does not [seem to be] clear [and] now it’s just submission of vital, if [give] wrong medication, how?’ [Patient G] |
| Patient/caregiver positively acknowledges instructions | Patient/caregiver acknowledges and demonstrates understanding of the healthcare provider’s instructions | ‘Thanks a lot and noted on this.’ [Patient H]  ‘I am thankful to have the [healthcare] team [and] to benefit from the hospital-at-home programme, [it was] very helpful to me and my family. [Patient I] |
| Delayed reply from patients/caregiver | Time elapsed for reply from patients/caregivers after office hours (After 6pm) | ‘Hi Mr [patient], awaiting your email address.’ [Doctor A] |
| Delayed reply from healthcare providers | Time elapsed for reply from healthcare providers after office hours (After 6pm) | ‘Sorry to revert back so late, we will discharge you tomorrow as planned.’ [Doctor A] |
| Healthcare provider responds to patient’s missed call | Healthcare provider receives the patient’s missed call and follows up with a text message | ‘Hi Mr [patient], we got a missed call from you. Is there anything we can help you?’ [Doctor A] |
| Informal tone by patient or caregiver | Short forms, smileys and exclamation marks used | ‘This is today’s test, fyi.’ |
| Formal tone by patient or caregiver | Salutations, proper sentences and punctuations used | ‘Hi [doctor], my dad agrees to the infusion. Please let me know the arrangements once you have the details, many thanks.’ [Caregiver F] |
| Informal tone by healthcare provider | Short forms, smileys and exclamation marks used | ‘Hi! Btw can you please help submit the consent form again but this time in your dad’s name? haha so sorry thank you!’ [Doctor C] |
| Formal tone by healthcare provider | Salutations, proper sentences and punctuations used | ‘Good morning, would like to remind [you] to submit today’s morning vitals please. Thank you.’ [Doctor A] |
